# Network Pharmacological Study on Mechanism of the Therapeutic Effect of Modified Duhuo Jisheng Decoction in Osteoporosis

**DOI:** 10.3389/fendo.2022.860649

**Published:** 2022-03-31

**Authors:** Xudong Huang, Zhou Zhou, Yingyi Zheng, Guoshuai Fan, Baihe Ni, Meichen Liu, Minghua Zhao, Lingfeng Zeng, Weiguo Wang

**Affiliations:** ^1^First Clinical Medical College, Shandong University of Traditional Chinese Medicine, Jinan, China; ^2^School of Basic Medical Science, Zhejiang University of Traditional Chinese Medicine, Hangzhou, China; ^3^The Second Affiliated Hospital of Guangzhou University of Chinese Medicine, Guangzhou, China; ^4^Affiliated Hospital of Shandong University of Traditional Chinese Medicine, Jinan, China

**Keywords:** osteoporosis, network pharmacology, TNF signaling pathway, molecular docking, Modified Duhuo Jisheng Decoction

## Abstract

**Background:**

Modified Duhuo Jisheng Decoction (MDHJSD) is a traditional Chinese medicine prescription for the treatment of osteoporosis (OP), but its mechanism of action has not yet been clarified. This study aims to explore the mechanism of MDHJSD in OP through a combination of network pharmacology analysis and experimental verification.

**Methods:**

The active ingredients and corresponding targets of MDHJSD were acquired from the Traditional Chinese Medicine System Pharmacology (TCMSP) database. OP-related targets were acquired from databases, including Genecards, OMIM, Drugbank, CTD, and PGKB. The key compounds, core targets, major biological processes, and signaling pathways of MDHJSD that improve OP were identified by constructing and analysing the relevant networks. The binding affinities between key compounds and core targets were verified using AutoDock Vina software. A rat model of ovariectomized OP was used for the experimental verification.

**Results:**

A total of 100 chemical constituents, 277 targets, and 4734 OP-related targets of MDHJSD were obtained. Subsequently, five core components and eight core targets were identified in the analysis. Pathway enrichment analysis revealed that overlapping targets were significantly enriched in the tumour necrosis factor-alpha (TNF-α) signaling pathway, an inflammation signaling pathway, which contained six of the eight core targets, including TNF-α, interleukin 6 (IL-6), transcription factor AP-1, mitogen-activated protein kinase 3, RAC-alpha serine/threonine-protein kinase, and caspase-3 (CASP3). Molecular docking analysis revealed close binding of the six core targets of the TNF signaling pathway to the core components. The results of experimental study show that MDHJSD can protect bone loss, inhibit the inflammatory response, and downregulate the expression levels of TNF-α, IL-6, and CASP3 in ovariectomized rats.

**Conclusion:**

The mechanism of MDHJSD in the treatment of OP may be related to the regulation of the inflammatory response in the bone tissue.

## Introduction

Osteoporosis (OP) is a systemic disease characterised by a decreased bone strength and damaged bone microstructure (1). OP increases bone brittleness, which is an important factor in causing fracture, and seriously affects human health and the quality of life of patients (2, 3). There are approximately 200 million patients with OP worldwide, and with the ageing population worldwide, its incidence rate has increased each year (4). Therefore, OP has become a global health concern. Presently, the treatment methods for OP mainly improve the clinical symptoms of patients by slowing bone loss, promoting bone formation, and inhibiting bone resorption, but these treatments have certain limitations (5, 6). Modified Duhuo Jisheng Decoction (MDHJSD) is derived from Duhuo Jisheng Decoction(DHJSD), a classic prescription for Bei Ji Qian Jin Yao Fang written by Sun Simiao in the Tang Dynasty. Contemporary studies have found that DHJSD has a good curative effect in the treatment of OP (7, 8). Wang Weiguo, deputy chief physician of The Affiliated Hospital of Shandong University of Traditional Chinese Medicine, combined with the clinical symptoms of OP patients, added and subtraction DHJSD, and finally obtain the self-drafted formula MDHJSD was composed of 10 herbs: including Cornus officinalis, Divaricate saposhniovia root, Gentiana macrophylla, Angelica pubescens, Spatholobus suberctu, Rhizoma Chuanxiong, Astragalus membranaceus, Eucommia ulmoides, TyTPae pollen and Rhizoma drynariae. Its reasonable curative effect has been clinically verified. However, the material basis and molecular mechanisms of MDHJSD in the treatment of OP are not yet elucidated.

**Figure 1:**
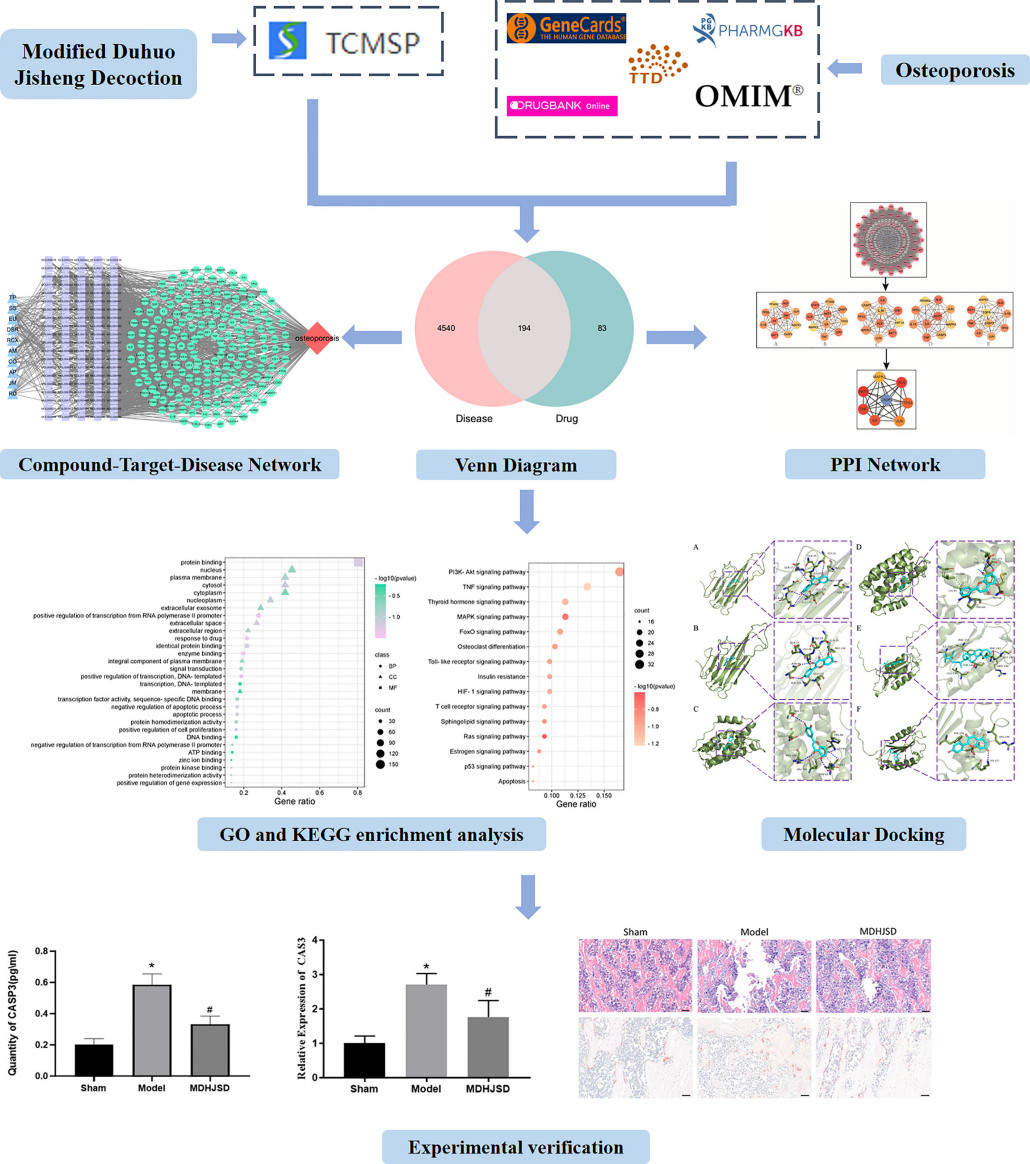
The research process. Compared with control operation group, *P < 0.05; Compared with the model group, ^#^*P* < 0.05.

Based on the theories of system biology and bioinformatics, network pharmacology explores the interaction between biomolecules and targets *in vivo* from the perspective of a systematic level and biological network, which can effectively predict the drug efficacy and its mechanisms (9, 10) to close the gap between modern medicine and traditional Chinese medicine. Therefore, it is widely used in research studies on traditional Chinese medicines. In this study, the network pharmacology method was used to integrate the information of drug active components, drug and disease targets, construct the network of the therapeutic effect of MDHJSD on OP, and reveal the material basis and mechanism of MDHJSD in the treatment of OP. The binding ability between the ligand and receptor was evaluated by molecular docking analysis, and some key genes were verified using animal experiments. The research process is shown in **Figure 1**.

## Materials and Methods

### Screening Effective Chemical Components and Action Targets of MDHJSD

We used the Traditional Chinese Medicine System Pharmacology (TCMSP) database (http://www.tcmspw.com/tcmsp.php) (11) of traditional Chinese medicine ingredients to search for MDHJSD constituent medicines (dogwood, fangfeng, qinjie, duhuo, jixueteng, chuanxiong, astragalus, eucommia, puhuang, and drynariae) to obtain all chemical and pharmacological data. According to the key parameters of oral bioavailability (OB) and drug-likeness (DL), the chemical components satisfying an OB ≥ 30% and a DL ≥ 0.18 were identified as being the active ingredients in MDHJSD. Additionally, the TCMSP database was used to screen the targets of the active ingredients of MDHJSD, and the drug targets were corrected and deduplicated in the Uniprot database (https://www.uniprot.org/) (12).

### Search for OP-Related Targets

A search using the Genecards, OMIM, Drugbank, CTD, and PGKB databases and using ‘osteoporosis’ as the keyword was conducted from which the OP-related targets were retrieved, and the final ones were obtained by taking the correlation score as the reference union set. We then drew a Wayne diagram to determine the intersections of the MDHJSD and OP targets.

### Construction of the Drug-Active Ingredient-Target Network

Microsoft Excel was used to sort the obtained drug active ingredients and intersection targets. The data were then imported into Cytoscape software, and a ‘drug-active ingredient-target’ network model was built, in which the nodes represent traditional Chinese medicines, ingredients, and targets, and in which the edges represent the relationship role between the three, using the number of associations of each node to calculate the ‘degree’ value.

### Construction of the Protein-Protein Interaction (PPI) Network and Screening of Key Targets

The intersection target between the MDHJSD active ingredient target and OP disease target was imported into the STRING database (https://www.string-db.org/cgi/input.pl), and ‘*Homo sapiens*’ was selected to obtain the PPI relationship (13). When importing the results into Cytoscape 3 7.2, the software constructed the interaction network diagram of ‘MDHJSD active ingredient OP target’, screened the common targets by using the closeness, degree, EPC, MCC, and MNC algorithms, and finally obtained the core target.

### Gene Ontology Biological Process (GO) and Kyoto Encyclopaedia of Genomes (KEGG) Pathway Enrichment Analyses

The David 6.8 (https://david.ncifcrf.gov/home.jsp) database was used to perform a GO enrichment analysis and KEGG pathway annotation analysis on the intersection targets of diseases and drugs (14), and bubble diagrams were drawn for the analysis of the biological processes of the first 15 KEGG pathways, which are significantly enriched, and the top 10 BP (biological pathways), CC (cell localization) and MF (molecular function) identified in the GO analysis.

### Docking of Effective Components With Key Target Genes and Molecules

In the drug-active ingredient-target network, the top five core compounds of MDHJSD in the treatment of OP were screened according to the ‘degree’ value, the 2D structure of the core compound was obtained in the PubChem database, and the 3D structure of the core target was obtained from the PDB database (15). After the compound was processed by Chem3D software, the analysis of molecular docking between the core compound and core target was carried out using AutoDock Vina software. The binding energy was used to evaluate the binding degree between the molecular compounds and targets. PyMOL software was used to visually display the docking results for those molecules exhibiting a high degree of combination.

### Establishment of the Experimental Animal Model of OP

Thirty-six female Sprague-Dawley rats aged 8–9 weeks and weighing 230–280 g were provided by Jinan Pengyue Experimental Animal Breeding Co., Ltd. Standard feed was made available at room temperature, and the rats were provided free access to drinking water and adaptive feeding for 5 days. The 36 rats were divided into 3 groups, with 12 rats in each group: the control, model, and treatment groups. Ovariectomies were performed in the model and treatment groups, and a similar incision without an ovariectomy was made in the control operation group, followed by suturing. Oophorectomy was followed by normal diet for 4 weeks, and MDHJSD administration was started after model establishment. The treatment group was administered 256.5 mg/kg/d MDHJSD by gavage, and the model and control operation groups were administered normal saline at the same dosage. After 3 weeks of administering continuous treatment, the abdominal aortic blood of the rats was collected under chloral hydrate anaesthesia, and the bilateral femurs were removed. All procedures were reviewed and approved by the experimental animal ethics committee of the Affiliated Hospital of Shandong University of Traditional Chinese Medicine (2020-48).

### Bone Morphometric Assessments

The rat femur was exfoliated and the muscle on the bone was removed. The femur was decalcified with 5% EDTA and embedded in paraffin blocks, and 5 mm sections were cut through a continuous incision in the sagittal plane. The sections were stained with hematoxylin and eosin (H&E) to observe the changes of bone trabecular and bone marrow cavity, and the activity of osteoclasts was detected by TRAP staining.

### Validation of Key Targets Through Real-Time Quantitative Reverse Transcription Polymerase Chain Reaction

When referring to relevant research findings (16), TNF-α, IL-6, and CASP3 were found to play an important role in the pathogenesis of OP, so we chose these three targets for inclusion in the experimental verification. Three key targets were verified by real-time quantitative reverse transcription polymerase chain reaction (qRT-PCR). The primers were designed and synthesized by the solid-phase phosphorous amide tri-ester method, and the sequences of the primers are shown in **Table 1**.

**Table 1:** The primer sequences used in the qRT-PCR analysis performed in this study.

Gene Name	BP	Forward primer (5′-3′)	Reverse primer (5′-3′)
GAPDH	70	TGAACGGGAAGCTCACTGGC	CATGTGAGATCCACGACGGACA
TNF-α	111	ATGGGCTCCCTCTCATCAGTTCC	CCTCCGCTTGGTGGTTTGCTAC
IL-6	110	ACTTCCAGCCAGTTGCCTTCTTG	TGGTCTGTTGTGGGTGGTATCCTCTAC
CASP3	136	GCGGTATTGAGACAGACAGTGGAAC	AACCATGACCCGTCCCTTGAATTTC

The rat femoral bone tissue (50 mg) was weighed, RNA was extracted with a Spark Easy Bone Tissue RNA Kit (Sparkjade, China), and a Spark Script II RT Plus Kit (Sparkjade, China) was used for the reverse transcription reaction. The two-step reaction procedure (Roche LightCycler480 II, Switzerland) was applied with pre-denaturation at 95°C for 10 min, and then 44 cycles of reaction at 95°C for 15 s and 60°C for 60 s were performed. Gene expression data were analysed using the 2^-ΔΔCt^ method.

### Enzyme Linked Immunosorbent Assay (ELISA) Analysis

Blood samples were taken and centrifuged for 30 min, from which the serum was obtained. According to the instructions of the ELISA kit, the optical density (OD) was measured using a microplate reader at a wavelength of 450 nm, and the protein expression levels of TNF-α (JYM0635Ra, ELISA LAB), IL-6 (JYM0646Ra, ELISA LAB) and CASP3 (ab281235, abcam) were quantified.

### Statistical Analysis

SPSS 21.0 software was used for the statistical analysis of the experimental data, and all data are expressed as the mean ± standard deviation. One-way analysis of variance was used for comparisons between multiple groups, the results of which were considered statistically significant at *P* < 0.05.

## Results

### Screening of MDHJSD Active Ingredients and Therapeutic Targets for OP

**Figure 2:**
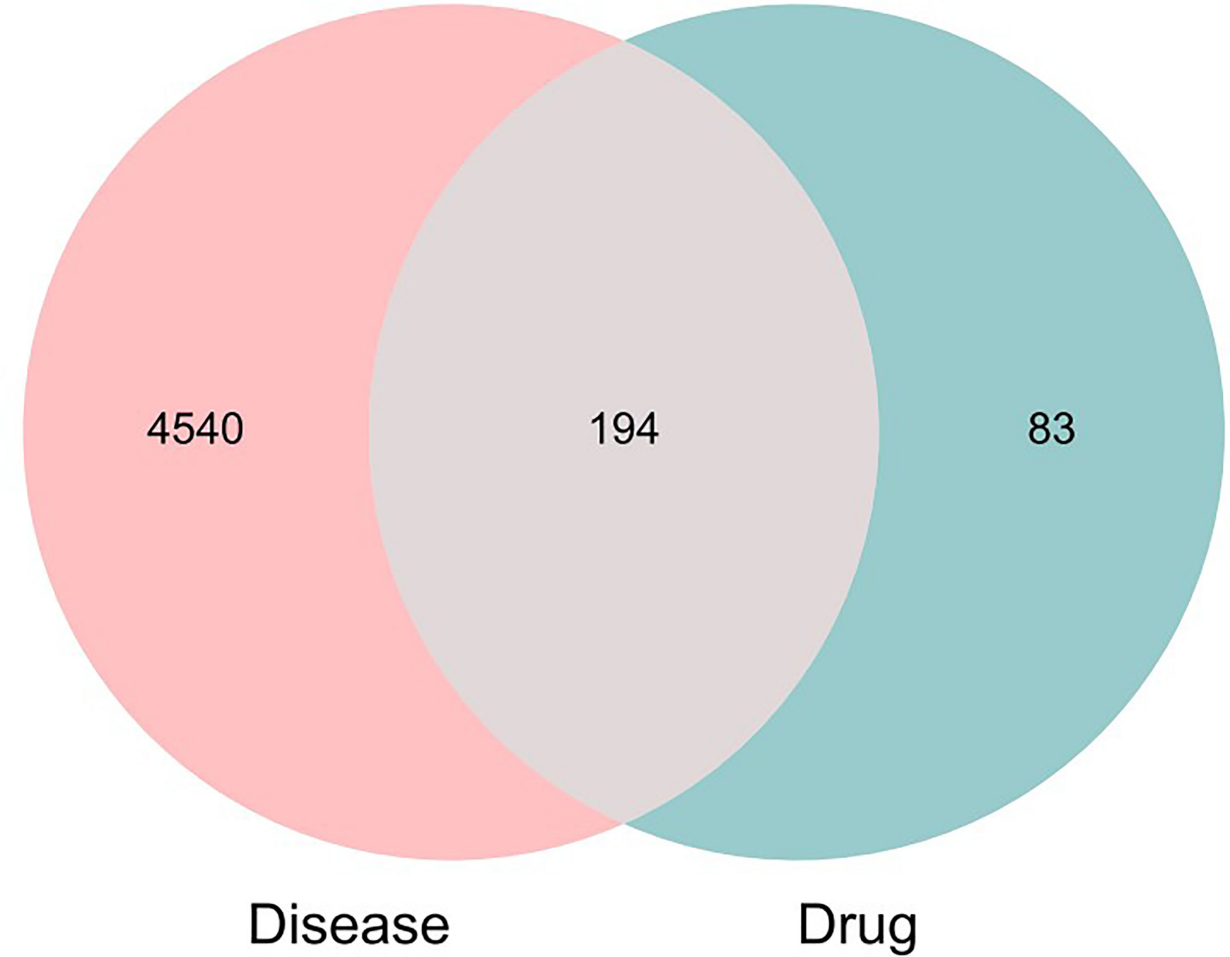
Venn diagram exhibiting the intersection of the MDHJSD targets and OP targets.

A total of 100 active ingredients from 10 formulations derived from MDHJSD (**Supplementary Appendix 1**) were retrieved from the TCMSP database according to the screening criteria of OB ≥ 30% and DL ≥ 0.18, and these corresponded to 277 targets. A total of 4734 targets related to OP treatment were obtained from five databases: the Genecards, OMIM, PharmGkb, DrugBank, and CTD. Using an online Venn diagram editing website (http://bioinformatics.psb.ugent.be/webtools/Venn/), we input the potential targets screened by OP and the targets that MDHJSD active ingredients act on and found 194 common genes (**Figure 2**), which are the potential target genes of MDHJSD in the treatment of OP (**Supplementary Appendix 2**).

### Construction of the Traditional Chinese Medicine-Active Ingredient-Target Network Diagram

**Figure 3:**
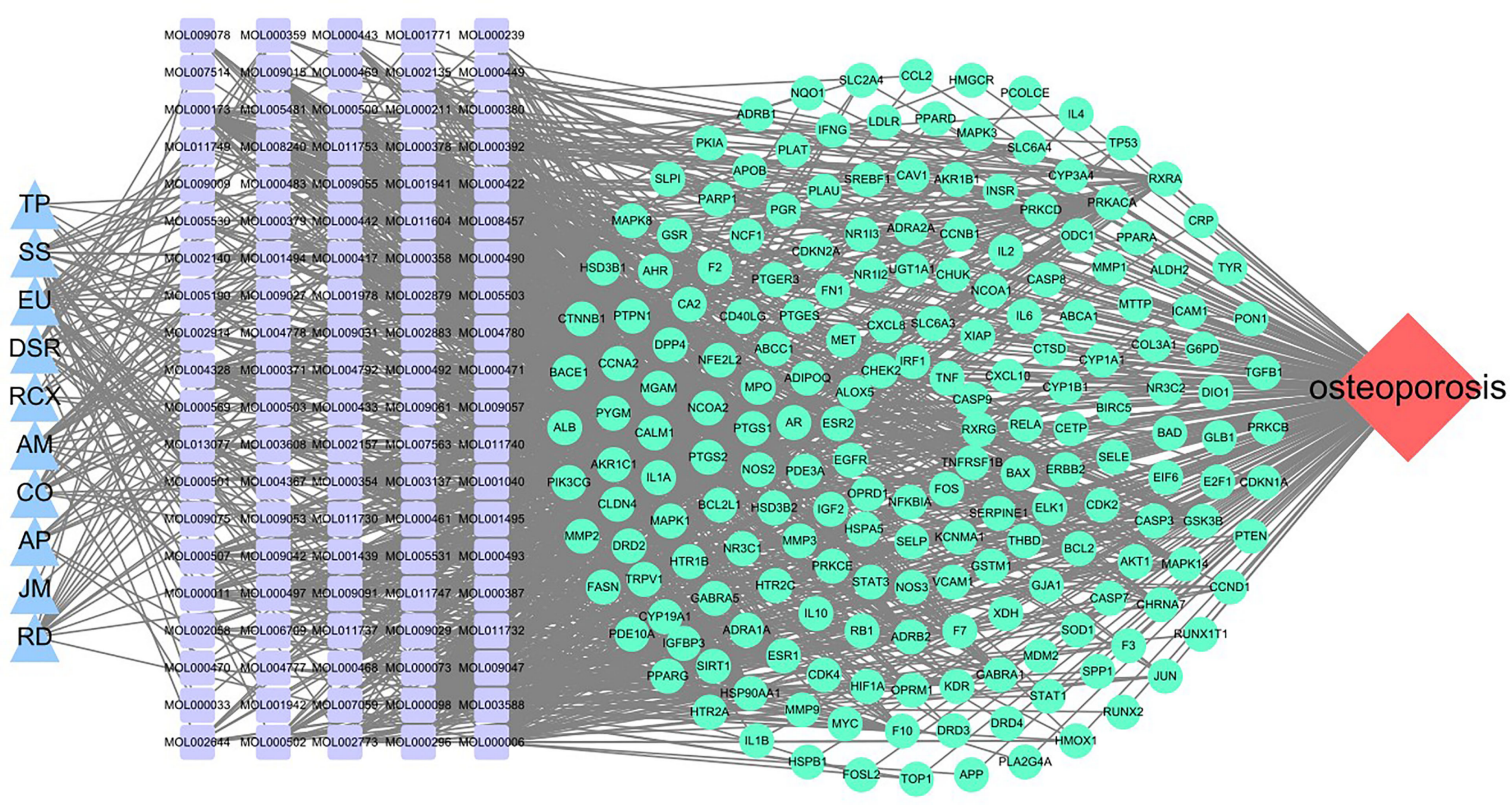
A medicine-ingredients-targets-disease network of four parts. Red: disease; Blue: medicine; Cyan: 194 potential common targets; Purple: active ingredients of MDHJSD.

The 10 traditional Chinese medicines, 100 active ingredients, and 194 intersecting target annotation relationships and classification obtained were imported into Cytoscape software, and the traditional Chinese medicine-active ingredients-target network diagram of MDHJSD in the treatment of OP was drawn (**Figure 3**). The square node in **Figure 2** represents the active ingredients, the triangle nodes represent the 10 traditional Chinese medicines of MDHJSD, and the circle nodes represent the targets related to the active ingredients and OP treatment. As shown in **Figure 2**, this network has 305 nodes and 92720 edges. Quercetin has the closest relationship with the therapeutic targets, with a total of 113 targets, followed by luteolin with 50 targets and kaempferol with 43 targets. It is considered that these active components may be the core components of MDHJSD in the treatment of OP.

### PPI Network Construction and Key Target Screening

**Figure 4:**
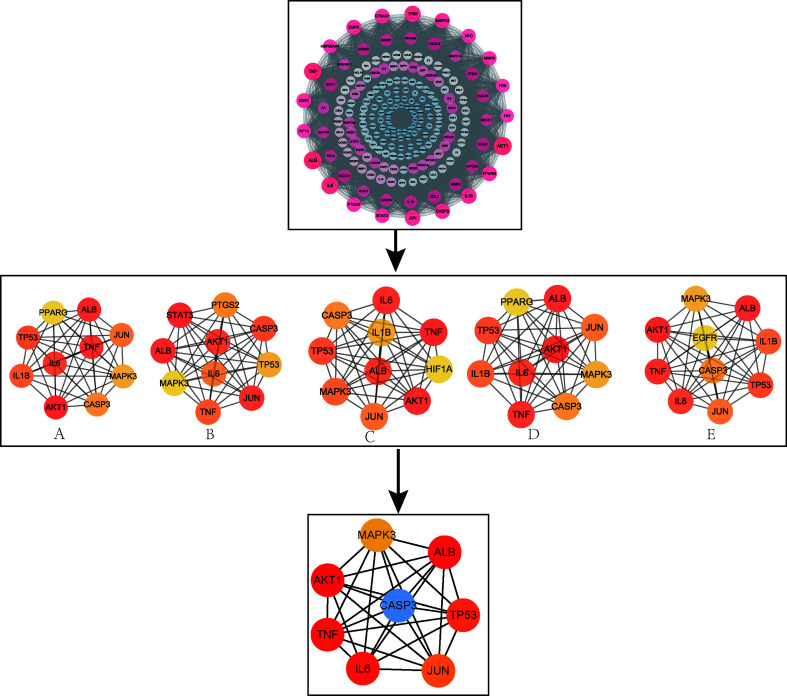
PPI network and screening of core targets. **(A)** MNC; **(B)** MCC; **(C)** EPC; **(D)** Degree; **(E)** Closeness.

The 194 intersection targets were imported into the STRING database to obtain the PPI relationship, and imported into Cytoscape 3.7.2 software to construct a PPI interaction map (17) (**Supplementary Appendix 3**). Finally, eight core targets using the closeness, degree, EPC, MCC, and MNC algorithms in the Cytohubba plug-in (**Figure 4**) were determined: IL-6, RAC-alpha serine/threonine-protein kinase (AKT1), mitogen-activated protein kinase 3 (MAPK3), CASP3, TNF, transcription factor AP-1 (JUN), cellular tumour antigen p53 (TP53), and albumin (ALB).

### GO Enrichment Analysis and KEGG Pathway Analysis of OP Targets in MDHJSD Therapy

**Figure 5:**
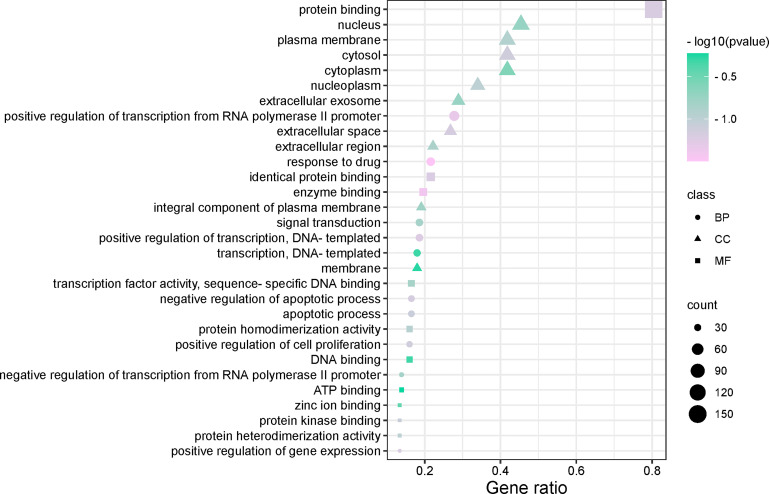
The top 10 remarkably enriched genes from the GO analysis (BP, CC, and MF).

We used the DAVID database and its expansion package to perform gene ontology (GO) enrichment analysis (*P* < 0.05) for the common targets between the drugs and disease. A total of 593 biological pathways, 70 cell localisations, and 121 molecular functions were obtained. GO enrichment analysis of disease-related genes was carried out with R software and its extension package, and the top 10 items of BP, CC and MF enrichment were visualized (**Figure 5**). The abscissa in the figure represents the gene enrichment rate (%), and the size of the icon represents the number of genes enriched in the process. The larger the icon, the more genes that were enriched. The icon colour represents the significance of enrichment, and the more purple, the more significant the enrichment. The results showed that BP was mainly related to the positive regulation of transcription from RNA polymerase II promoter, response to drug, transcription of DNA templates, etc. CC mainly included the nucleus, cytosol, plasma membrane, cytoplasm, etc. MF mainly included the protein binding, identity protein binding, enzyme binding, etc.

**Figure 6:**
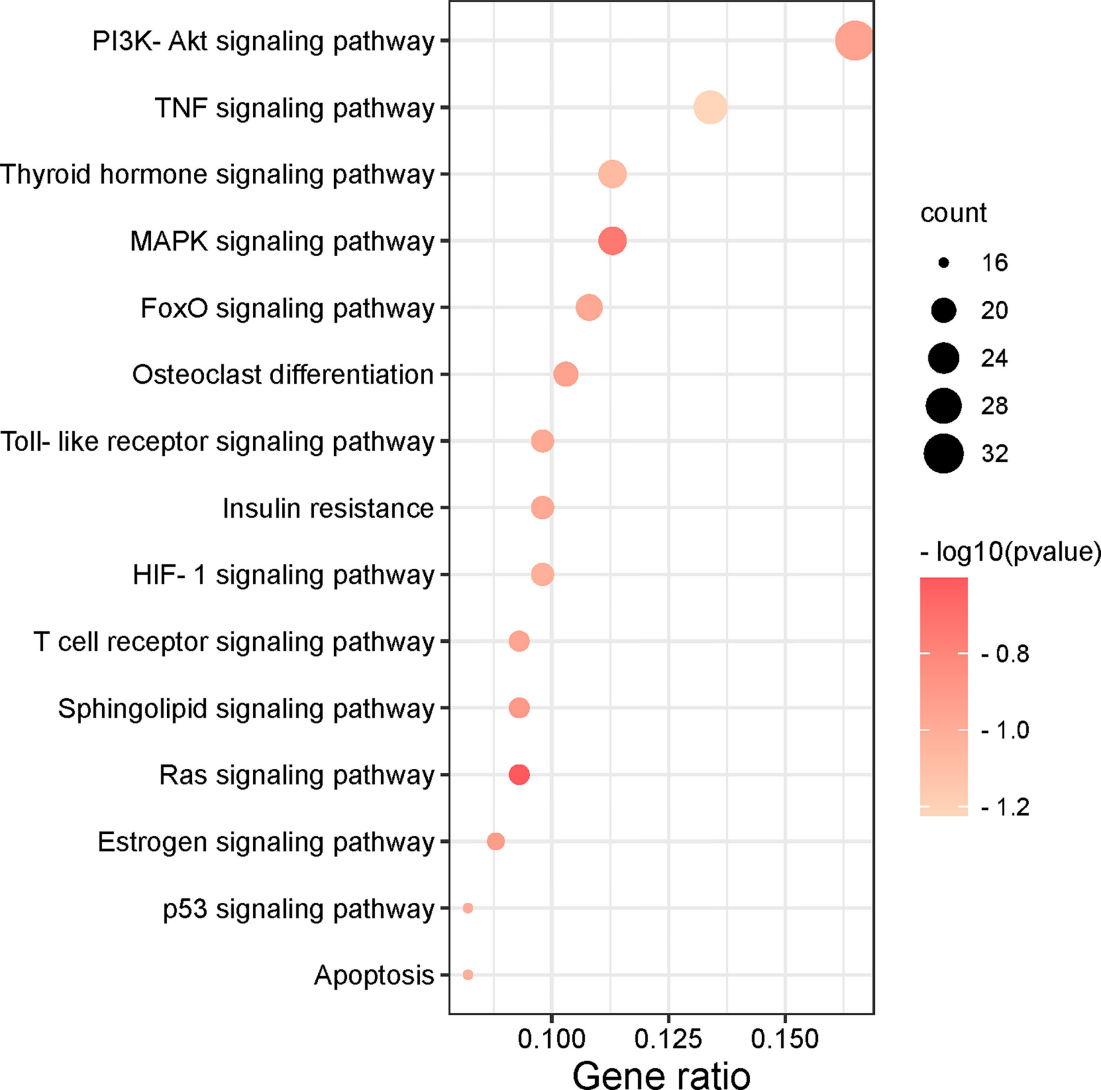
The top 15 remarkably enriched signaling pathways from the KEGG analysis for the signaling pathway of potential target genes of MDHJSD in OP.

To further understand the potential pathway of MDHJSD in the treatment of OP, we performed KEGG channel enrichment analysis and obtained 125 signal channels (*P <* 0.05). Fifteen pathways related to OP were screened, including the PI3K-Akt, TNF, thyroid hormone, and MAPK signaling pathways. The channels are then visualised according to the number of key genes in these channels (**Figure 6**). The results showed that the top three enriched signaling pathways were the PI3K-Akt, TNF, and thyroid hormone signaling pathways. The TNF signaling pathway is constituted by six of the eight core targets: *TNF-α, IL-6, JUN, MAPK3, AKT1*, and *CASP3*, so this pathway may mediate the anti-OP effect of MDHJSD.

### Verification of Molecular Docking

**Figure 7:**
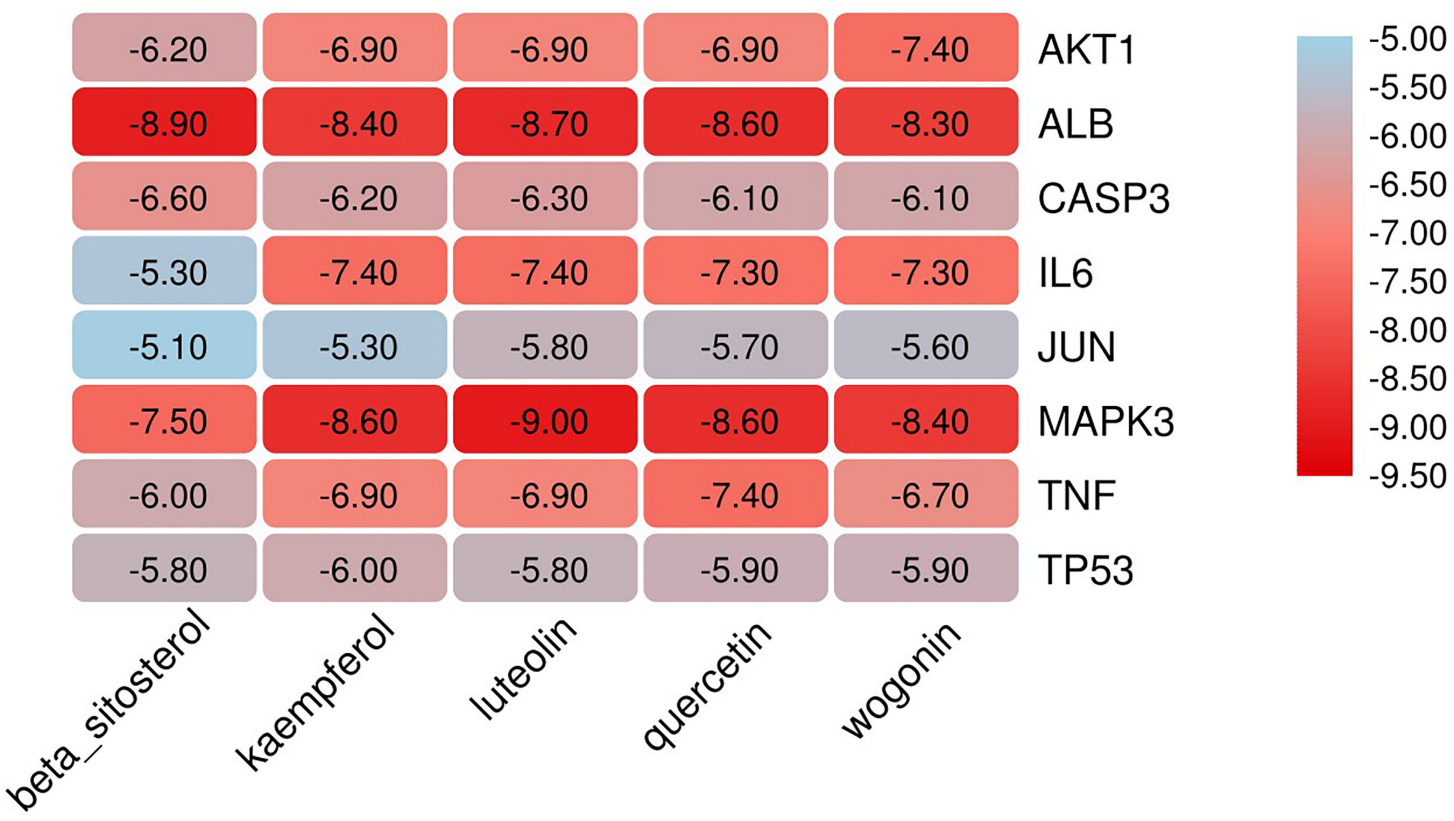
Docking heat-map of the core components and core targets. Affinity: kcal/mol.

**Figure 8:**
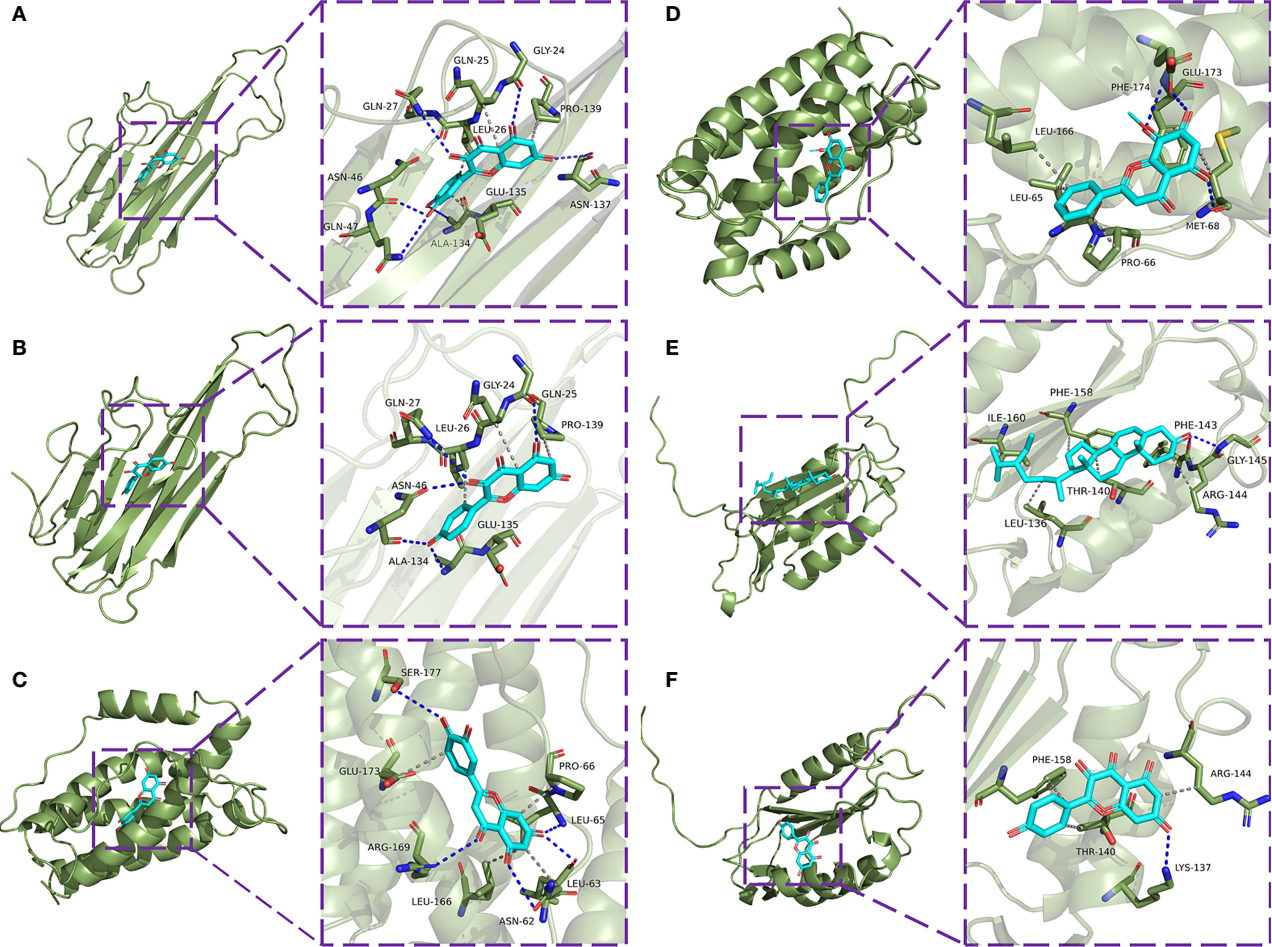
Molecular docking model diagram. **(A)** Molecular docking of TNF-α with quercetin. **(B)** Molecular docking of TNF with kaempferol. **(C)** Molecular docking of IL-6 with luteolin. **(D)** Molecular docking of IL-6 with wogonin. **(E)** Molecular docking of CASP3 with beta-sitosterol. **(F)** Molecular docking of CASP3 with kaempferol.

The eight core targets screened in the PPI network analysis were associated with the top five active components of ‘degree’ in the drug-active ingredient-target network (**Table 2**). Considering the binding energy as an evaluation of docking degree, it was considered that the binding energy ≤-5 kcal/mol indicates that they can be combined, and ≤-7 kcal/mol indicates that they have a good binding ability (18). We drew the hot spot diagram of the docking results (**Figure 7**). The colour of the square in the diagram is biased to red, indicating that the binding energy between the corresponding component and the target protein is low and the binding degree is high. The colour is blue, indicating that the binding energy between the corresponding component and the target protein is high and the binding degree is poor. It can be observed from **Figure 6** that the core components and core targets have a good binding ability. Six pairs of binding relationships were selected: TNF and quercetin, TNF and kaempferol, IL-6 and luteolin, IL-6 and wogonin, CASP3 and beta-sitosterol, and CASP3 and kaempferol, which were visualized by PyMOL software (**Figure 8**).

**Table 2:** Top 5 active ingredients in the ‘degree’ analysis.

NO	Molecule ID	Molecule name	Degree
1	MOL000098	quercetin	116
2	MOL000006	luteolin	51
3	MOL000422	kaempferol	47
4	MOL000173	wogonin	39
5	MOL000358	beta-sitosterol	32

### Effect of MDHJSD on Morphology of Femur Bone in Rats

**Figure 9:**
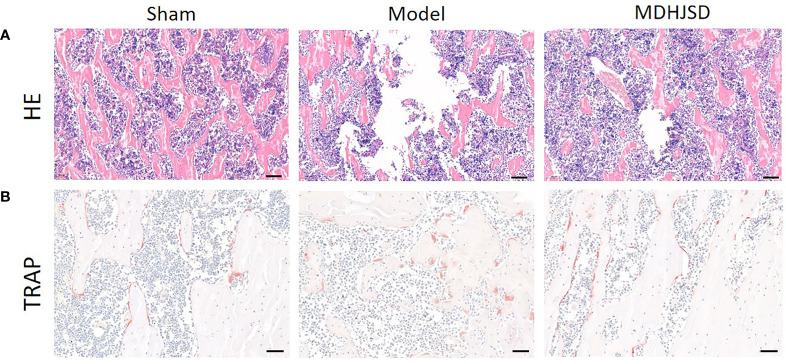
The pathological sections of femur of rats in each group at the end of the 3th week; **(A)** H&E staining: Scale bar = 100μm, 100×; **(B)** TRAP staining: Scale bar = 50μm, 200×.

H&E staining (**Figure 9A**) showed that trabecular bone in the Sham group were evenly distributed and in normal morphology, while trabecular bone in the Model group were sparse and disorderly with wider spacing. Compared with Model group, the distribution of trabecular bone in MDHJSD group was more uniform, and the density of trabecular bone was roughly restored. TRAP staining (**Figure 9B**) showed that the number of osteoclasts was reduced in MDHJSD group compared with Model group, indicating that MDHJSD could inhibit osteoclasts proliferation in oophorectomy rats.

### MDHJSD Effect on mRNA Expression Levels of Key Targets

**Figure 10:**
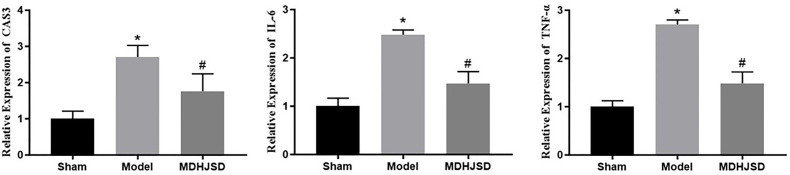
Effect on key target mRNA expression levels. Compared with control operation group, *P < 0.05; Compared with the model group, ^#^*P* < 0.05.

The qRT-PCR results showed (**Figure 10**) that, compared with the control-operated group, the mRNA expression levels of TNF-α, IL-6, and CASP3 in the bone tissue of the model group and the treatment group were significantly increased (*P* < 0.05). Compared with the model group, the mRNA expression levels of TNF-α, IL-6, and CASP3 in the bone tissue of the treatment group were significantly decreased (*P* < 0.05). The results showed that MDHJSD could reduce the mRNA expression levels of TNF-α, IL-6, and CASP3 in the bone tissue of the rats induced with OP.

### Related Protein Expression

**Figure 11:**
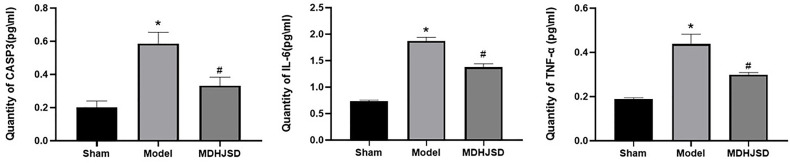
Expression levels of the related proteins. Compared with control operation group, *P < 0.05; Compared with the model group, ^#^*P* < 0.05.

The ELISA results showed (**Figure 11**) that compared with the rats in the control-operated group, the expression levels of TNF-α, IL6, and CASP3 in the serum of the model group and the treatment group were significantly increased (*P* < 0.05). Compared with the model group, the expression levels of the TNF-α, IL-6, and CASP3 proteins in the treatment group were significantly decreased (*P* < 0.05), and the differences for which were statistically significant. The ELISA results were consistent with the qRT-PCR results.

## Discussion

The process of bone remodelling occurs throughout the human body and is a coordinated process of repairing small fractures and maintaining bone mass. Under normal circumstances, bone formation and bone resorption always maintain homeostasis. When bone resorption is faster than bone formation, the balance of bone remodelling is disrupted (19), resulting in a decrease in bone content per unit volume and a decrease in bone density, which eventually leads to the development of OP (20). As the pathogenesis of OP is the result of a combination of factors and multiple targets and pathways are involved in the pathological process (21), the current therapeutic efficacies of single-target drugs are lower than expected. Traditional Chinese medicine has the advantages of having multiple components and multiple targets in the treatment of diseases (22), so it has a high clinical value in the treatment of OP. In previous clinical trials, we found that MDHJSD can clearly improve the clinical symptoms of patients with OP and reduce the incidence of fractures (especially elderly patients), but its mechanism of action has not yet been elucidated. It has good prospects for use in the treatment of OP and provides more therapeutic strategies for the treatment of OP. This study aimed to analyse the mechanism of action of MDHJSD in the treatment of OP through network pharmacology and to verify the identified mechanism using animal experiments.

One hundred active components and 277 corresponding targets of MDHJSD were retrieved, and 4734 OP-related targets were obtained, including 194 overlapping targets. Finally, five active components, including quercetin, luteolin, kaempferol, wogonin, and beta-sitosterol, were screened according to the degree value. The PPI network was analysed by the Cytohubba plug-in, and the final eight core genes were obtained: IL-6, AKT1, MAPK3, CASP3, TNF, JUN, TP53, and ALB. According to the results of the pathway enrichment analysis, it was found that overlapping targets were significantly enriched in the PI3K-Akt, TNF, and thyroid hormone signaling pathways. In the molecular docking verification, the results showed that TNF-α, IL-6, and CASP3 had good docking activity with the five core active components, indicating that they may be the potential active components and targets of MDHJSD in the treatment of OP.

As far as we know, the inflammatory response plays an important role in the occurrence and development of OP. Many inflammatory factors play a key role in OP, which can not only lead to obstacles in bone formation but also strengthen bone resorption activities and cause the imbalance of bone reconstruction (23, 24). Clinical observation shows that systemic osteoporosis occurs simultaneously with periodic systemic inflammation, and local osteoporosis occurs simultaneously with local inflammatory areas (25). Pasco’s study on osteoporosis found that the risk of fracture increased 24-32% for each SD increase in CRP levels in older women (26). Ding and collaborators studied 168 men and women with an average age of 63 years and found that increased bone loss was associated with higher levels of systemic inflammation during a 3-year follow-up (27). Therefore, some scholars believe that postmenopausal inflammatory factors, such as TNF -α and IL-6, are the factors leading to accelerated bone mineral density (BMD) decline (28, 29).

TNF-α is an important mononuclear inflammatory factor that can degrade organic bone by inhibiting extracellular matrix deposition. Relevant studies found that TNF-α can not only inhibit the transformation of mesenchymal stromal cells (MSCs) to osteoblasts by regulating the expression of Runx2 but also inhibit the expression of osterix (OSX; Sp7) and affect the development and maturation of osteoblasts (30, 31). In addition, TNF-α can also regulate the expression of insulin like growth factor I (IGF-I), inhibit the differentiation of osteoblasts, affect bone formation, directly or indirectly promote the production of macrophage colony stimulating factor, and accelerate the bone resorption process in osteoclasts (32, 33). In addition, abundant evidence that TNF-α can induce osteoblast apoptosis, disrupt the balance of bone remodeling, and lead to loss of bone content (34, 35). IL-6 is a multifunctional inflammatory factor that plays an important role in a variety of biological bone activities (36, 37). Experimental studies have found that IL-6 can induce osteocyte differentiation, promote osteoclast formation, enhance osteoclast activity, accelerate bone resorption, and aggravate the degree of OP (38–40). Thus, IL-6 plays an important role in the occurrence of OP.

The aim of the experimental verification in this study was to construct an OP animal model by oophorectomy. The results showed that MDHJSD could significantly reduce femoral TNF-α in ovariectomized rats. The relative mRNA expression levels of IL-6 and CASP3 and the expression levels of the related proteins in serum osteoblasts protected from TNF-α induced apoptosis. MDHJSD may protect bone loss in ovariectomized rats by regulating TNF signaling channels.

## Study Limitations

Due to the limited time and funds of this study, the relationship between MDHJSD dose and efficacy in the treatment of OP was not studied. In addition, this study was a preliminary exploratory experiment to explore the mechanism of MDHJSD improving OP, and the mechanism of MDHJSD treating OP was not fully clarified. Therefore, in the following work, the research group will further discuss the above problems, and provide a certain scientific basis for the wide application of MDHJSD in clinical practice. Anyway, we still found that MDHJSD can significantly improve the progress of OP, and its mechanism may be related to the inhibition of inflammatory response, which will provide some scientific reference for further mechanism research.

## Conclusions

In conclusion, this study screened the key targets and pathways involved in the anti-OP effect by MDHJSD through network pharmacology, further using molecular docking analysis to clarify the mechanism of its therapeutic effect, and finally verified that MDHJSD can significantly reduce TNF-α in bone tissue through animal experimental research in the mRNA expression levels of IL-6 and CASP3. Therefore, we speculated that MDHJSD can effectively treat OP by inhibiting the inflammatory response, reducing bone resorption, and promoting bone shape.

## Data Availability Statement

The original contributions presented in the study are included in the article/**Supplementary Material**. Further inquiries can be directed to the corresponding authors.

## Ethics Statement

The animal study was reviewed and approved by the experimental animal ethics committee of the Affiliated Hospital of Shandong University of traditional Chinese medicine (2020-48).

## Author Contributions

WW and LZ conceived this project. XH and ZZ designed the study,acquisition of data, analysis and interpretation of data. GF, YZ, and BN searched the literature data. XH and ZZ drafted the manuscript. ML and MZ revision of the manuscript. All authors contributed to the article and approved the submitted version.

## Funding

Shandong Provincial Scientific and Technological Innovation Program for Medical Workers (No.202014), Science and Technology Project of Shandong Health Science and Technology Association (No.2017004).

## Conflict of Interest

The authors declare that the research was conducted in the absence of any commercial or financial relationships that could be construed as a potential conflict of interest.

## Publisher’s Note

All claims expressed in this article are solely those of the authors and do not necessarily represent those of their affiliated organizations, or those of the publisher, the editors and the reviewers. Any product that may be evaluated in this article, or claim that may be made by its manufacturer, is not guaranteed or endorsed by the publisher.

## Abbreviations

OP, Osteoporosis; MDHJSD, Modified Duhuo Jisheng Decoction; DHJSD, Duhuo Jisheng Decoction; TNF-α, Tumor necrosis factor-alpha; IL-6, Interleukin 6; CASP3, Caspase-3; OB, Oral bioavailability; DL, Drug-likeness; PPI, Protein-protein interaction; KEGG, Kyoto Encyclopaedia of Genomes; BP, Biological pathways; CC, Cell localization; MF, Molecular function; CO, Cornus officinalis; DSR, Divaricate saposhniovia root; GM, Gentiana macrophylla; AP, Angelica pubescens; SS, Spatholobus suberctu; RCX, Rhizoma Chuanxiong; AM, Astragalus membranaceus; EU, Eucommia ulmoides; TP, TyTPae pollen; RD, Rhizoma drynariae; BMD, Bone mineral density; CRP, C-reactive protein.
