# Supplementary material for: Network Pharmacological Study on Mechanism of the Therapeutic Effect of Modified Duhuo Jisheng Decoction in Osteoporosis
Source: Front Endocrinol (Lausanne). 2022 Mar 31;13:860649. doi: 10.3389/fendo.2022.860649 (PMC9008312; doi:10.3389/fendo.2022.860649)
Supplement: Supplementary file 1 [file DataSheet_1.zip › Supplementary Appendix 3.DOC]

Protein–protein interaction (PPI) network

A.Closeness；B.Degree；C.EPC；D.MCC；E.MNC
